# Supplementary material for: Development of a neural network model to predict the presence of fentanyl in community drug samples
Source: PLoS One. 2023 Jul 13;18(7):e0288656. doi: 10.1371/journal.pone.0288656 (PMC10343080; doi:10.1371/journal.pone.0288656)
Supplement: Attachment — Submitted filename: Response to Reviewers.docx [file pone.0288656.s001.docx]

**Response to Reviewer 1:**

We thank Reviewer 1 for their succinct comments about our manuscript. We have responded to Reviewer 1’s individual comments using numbered bullets below.

1. We thank Reviewer 1 for the comment regarding the need for grammatical corrections. The updated version of the manuscript has received thorough proofreading and we feel it is free of grammatical errors.
2. We thank Reviewer 1 for their suggestion that the “statistical analysis needs more details.” In light of this comment, we have included the following information that further describes the statistical analysis portion of methods:

**In assessing our model's performance, we utilized the prediction value which is a continuous variable ranging from 0 to 1. We applied a rounding procedure to transform this continuous output into a binary classification to align with our label values (the results from the corresponding fentanyl test strip for that sample). Specifically, prediction values greater than or equal to 0.5 were rounded up to 1, indicating a positive fentanyl result, while values less than 0.5 were rounded down to 0, indicating a negative result. These rounded prediction values were then compared against our label values, which represent the binary outcomes of the fentanyl test strip.** **Using these results,** we calculated several diagnostic metrics, including F1 score, accuracy, precision, recall, and area under the receiver-operating characteristic curve (AUROC). An F1 score describes a model’s accuracy, taking the precision (correctly identified positives among test positives) and recall (correctly identified positives among true positives) into account.

3. We thank Reviewer 1 for raising the issue of ambiguity regarding how we speak of file types. We have removed reference to “FTIR spectra files” and replaced it with the following:

…(jcamp-dx) encoded **FTIR data** files were converted to data tables…

4. We apologize to Reviewer 1 and the Editor that we are unable to determine what Reviewer 1 meant by the comment, “4. FTIR peaks which 4. Missing the main peaks indicated this space.” We hope that with the revisions in the updated manuscript, this comment has been sufficiently addressed.

5. We thank Reviewer 1 for highlighting the need to ensure consistency in reference style. As a part of our proofreading process, we have ensured consistency in reference formatting.

**Response to Reviewer 2:**

We thank Reviewer 2 for their time and generous comments regarding our manuscript. Reviewer 2 commented that “the manuscript is a technically sound piece of scientific research” and raised no concerns or need for any edits.

**Response to Reviewer 3:**

We thank Reviewer 3 for their comments about our manuscript. We have responded to Reviewer 3’s numbered comments below:

1. We appreciate Reviewer 3’s comments about the abstract being too long. While the abstract length is within the limit outlined in the *PLOS ONE* submission guidelines, we are happy to shorten it if the Editor deems it necessary.
2. We thank Reviewer 3 for noting additional keywords are beneficial. We do, however, feel that the keyword “harm reduction” is relevant to this article. We have added the additional keywords of “deep learning” and “FTIR spectroscopy.”
3. We thank Reviewer 3 for their comment regarding the use of the term “drug checking” in the manuscript. Drug checking is the internationally recognized term to refer to the harm reduction intervention of analyzing illicit drugs to determine what they contain before consumption. We feel the use of this specific term is adequately defined with supporting references in the manuscript:

In response, drug checking services have been implemented as an important harm reduction intervention for people who use drugs to chemically analyze their substances and receive fact-based information and consultation regarding the compounds detected in their sample [4–6]. Not a new concept, the vast majority of drug checking services have previously focused on drugs used in party and festival settings (e.g., psychedelics, stimulants) [7,8], and less is known about drug checking in the context of the synthetic opioid-driven overdose epidemic. While limited, recent studies have started to show a positive impact of drug checking, including increased engagement in overdose risk reduction practices following the use of the service in some communities [5,9–11].

4. Unfortunately, we are unable to discern which specific sentence Reviewer 3 is referencing in their 4^th^ comment, as the line numbers referenced do not contain sentences, but instead are the manuscript keywords. We apologize to Reviewer 3 and hope that during the revisions stage their concern has been adequately addressed otherwise.
5. We thank Reviewer 3 for their comment regarding the clarity of the use of the term “claimed” to refer to loss of life. We have amended the sentence to the following:

By the time a public health emergency was declared in 2016, a significant number of lives had already been **lost to illicit drug toxicity**, largely due to the widespread presence of fentanyl and other novel synthetic opioids in the unregulated drug supply [29].

6. We appreciate Reviewer 3’s comment about the clarification needed on how fentanyl-positive samples were obtained. The manuscript has been updated to provide further detail:

Further details about the drug checking service and the use of these technologies, which have been operating since October 2017, have been described elsewhere in detail [15,20]. **Briefly, individuals in harm reduction sites anonymously provided an approximately 5 mg drug sample to a trained drug checking technician and information about what they believed the sample to contain [34]. The drug sample was then analyzed with FTIR and immunoassay strips, with results being immediately available to the person who provided the sample. Results from FTIR analysis include compounds detected in abundant amounts (typically those 5% by weight and above), including both active ingredients (e.g., fentanyl, heroin, methamphetamine) and inert cutting agents (e.g., mannitol, inositol, lactose) [6,17,20].**

7. We thank Reviewer 3 for their comment about details of FTIR analysis and how it does not give information about quantity of specific compounds. To address this, we have added information to the methods section about the results generated by FTIR analysis at point of care:

**The drug sample was then analyzed with FTIR and immunoassay strips, with results being immediately available to the person who provided the sample. Results from FTIR analysis include compounds detected in abundant amounts (typically those 5% by weight and above), including both active ingredients (e.g., fentanyl, heroin, methamphetamine) and inert cutting agents (e.g., mannitol, inositol, lactose) [6,17,20].**

8. We thank Reviewer 3 for the comment about how the manuscript would benefit from further description about the artificial neural network. To address this comment, we have restructured and added additional text to the “Prediction methods and model selection” subsection of the methods section:

**The goal of the study was to use anonymous, point-of-care drug checking data obtained from FTIR spectra to predict the presence of fentanyl and related analogues in drug samples. We implemented an artificial neural network-based model, which was composed of six layers in the following order (Fig 1):**

**1) an input layer**

**2) a one-dimensional convolution layer with eight filters, a kernel size of 12, and a rectified linear unit (ReLU) activation function**

**3) a dropout layer with a 50% dropout rate**

**4) a one-dimensional max pooling layer with a pool size of two**

**5) a dense layer with output dimension 32 and a ReLU activation function**

**6) a dense output layer with output dimension one and a sigmoid activation function.**

**The model was trained on 80% of the data and 20% of the data was reserved for testing the model's accuracy. At each epoch, the accuracy (i.e., number of correct predictions among total number of predictions) was measured on the test set, and the model with the highest accuracy in the test set was kept.**

9. We thank Reviewer 3 for their suggestion about how to improve Fig 2 (now Fig 3). We feel that the ROC curve presented in Fig 2 sufficiently describes the performance classification characteristics that it seeks to depict. If the Editor feels that further description of characteristics is warranted, we can address this comment in another fashion.
10. We thank Reviewer 3 for suggesting how the conclusions should be separated from the discussion section. We have separated the two sections in the manuscript.
11. We thank Reviewer 3 for their comment regarding a graphical representation of a full factorial design. We have included a graphical representation of the model (Fig 2) and we hope that this graphic helps illustrate the final model developed over the course of the present study.

**Response to Reviewer 4:**

1. We thank Reviewer 4 for identifying previous research involving machine learning to identify fentanyl in various forms. Unfortunately, these articles are not related to the identification of fentanyl in illicit drug samples, but instead one is related to biological fluids and the other is related to chemical structure analysis. We feel these previous articles do not detract from the novel findings of our research, as it remains the first in published literature to describe the use of deep learning to detect fentanyl in illicit drug samples directly.
2. We thank Reviewer 4 for sharing their expertise regarding the use of different (and more sensitive) technologies to detect fentanyl in biologic samples. We have included new text that describes the advantages and disadvantages of using such technologies in a point-of-care drug checking setting to address the unregulated drug supply. We have also provided further information on the FTIR.

Drug checking can be conducted using a range of technologies, offering both benefits and challenges in terms of accuracy, timing, cost, portability, ability to detect a range of compounds, preparation, and required human resources [4,6,12,13]. For example, gas chromatography/mass spectrometry (GC-MS) is highly sensitive, but is expensive, time-consuming, and requires a trained laboratory technician to operate the machine [6,14]. In contrast, immunoassay strips are inexpensive, easy to use, portable, and can provide results in less than five minutes, but they are unable to detect a range of compounds and can only produce qualitative results (i.e., presence, absence) [15,16]. Fourier-transform infrared (FTIR) spectroscopy is being implemented across North America for drug checking given the ease and speed of sample preparation and analysis, portability, and ability to detect a range of compounds [15,16]. However, recent validation studies have indicated that FTIR spectroscopy can only detect compounds above a certain limit; for fentanyl and some fentanyl analogues, that limit is 3-10% quantity by weight, depending on the setting and the presence of specific cuts and buffs [16,17]. Given that some analogues (e.g., carfentanil) can cause toxicity at very low concentrations [18,19], failing to detect these compounds when present is a substantial concern when using FTIR spectroscopy on its own.

…

Further details about the drug checking service and the use of these technologies, which have been operating since October 2017, have been described elsewhere in detail [15,20]. **Briefly, individuals in harm reduction sites anonymously provided an approximately 5 mg drug sample to a trained drug checking technician and information about what they believed the sample to contain [34]. The drug sample was then analyzed with FTIR and immunoassay strips, with results being immediately available to the person who provided the sample. Results from FTIR analysis include compounds detected in abundant amounts (typically those 5% by weight and above), including both active ingredients (e.g., fentanyl, heroin, methamphetamine) and inert cutting agents (e.g., mannitol, inositol, lactose) [6,17,20].**

As well, we have described in more detail the use of FTIR spectroscopy more generally, in addition to including a new figure that depicts a typical FTIR spectrum (per Reviewer 4’s fourth comment).

3. We thank Reviewer 4 for their comment about providing further details on the methods to allow for replicability of the manuscript. We feel the manuscript, particularly with the new amendments in the course of the revisions process, have provided sufficient instruction to *PLOS ONE* readers should they wish to replicate the study, including new text and restructuring the “Prediction methods and model selection”. Upon further review, should the Editor feel further enhancements are necessary, we defer to their decision.
4. We agree with Reviewer 4’s comment that the manuscript lacks description of the structure of the fentanyl molecule and what an FTIR spectrum is and looks like. To address this issue, we have added an additional figure to the manuscript that depicts what a typical FTIR spectrum of a sample in our analysis looks like. The following text has also been added to the manuscript to more thoroughly describe FTIR spectra.

**An illustrative example of what a drug checking technician analyzes (and attempts to determine if fentanyl is present) is displayed in Fig 1.**

[Figure caption] **Fig 1. Sample drug checking Fourier-transform infrared spectra.** Drug checking samples (blue) are overlaid with the fentanyl hydrochloride spectrum (black). The drug sample in A is an illicit opioid mixture containing fentanyl, caffeine, and mannitol. The drug sample in B is methamphetamine with no fentanyl present. The red arrows indicate characteristic peaks of fentanyl hydrochloride that are recognizable when present in drug mixtures.
